# Supplementary material for: High-Dosage NMN Promotes Ferroptosis to Suppress Lung Adenocarcinoma Growth through the NAM-Mediated SIRT1–AMPK–ACC Pathway
Source: Cancers (Basel). 2023 Apr 23;15(9):2427. doi: 10.3390/cancers15092427 (PMC10177531; doi:10.3390/cancers15092427)
Supplement: Supplementary file 1 [file cancers-15-02427-s001.zip › cancers-2270516-supplementary.pdf]

High-Dosage NMN Promotes Ferroptosis to Suppress Lung Adenocarcinoma Growth through the NAM-Mediated SIRT1–AMPK–ACC Pathway

Supplementary figures

Supplementary Figure S1. High-dosage NMN enhanced cytotoxicity to lung adenocarcinoma cells than normal cells

Supplementary Figure S2. NMN accelerated lung adenocarcinoma cells death under ferroptosis inducers treatment

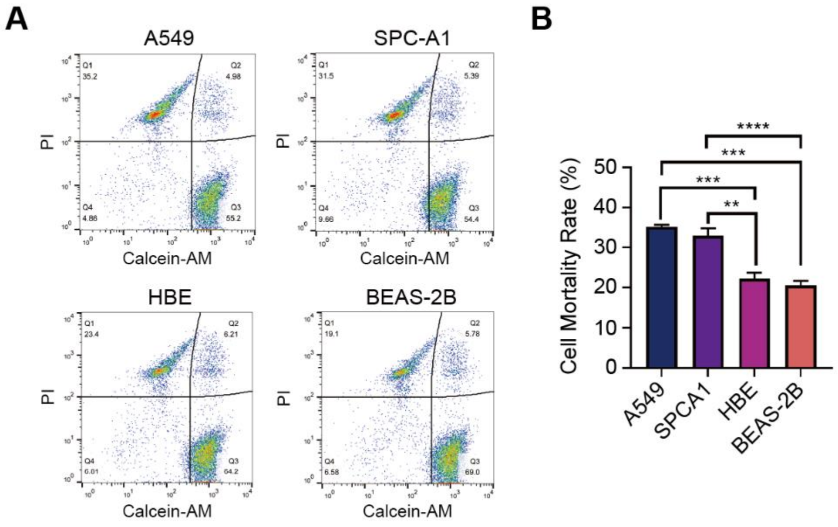

Supplementary Figure S1. High-dosage NMN enhanced cytotoxicity to lung adenocarcinoma cells than normal cells

A, FACS of A549, SPCA1, HBE, and BEAS-2B cells after high-doses NMN treatment. The representative images of FACS analysis. **B,** Quantitation of cell mortaility (*n* = 3).

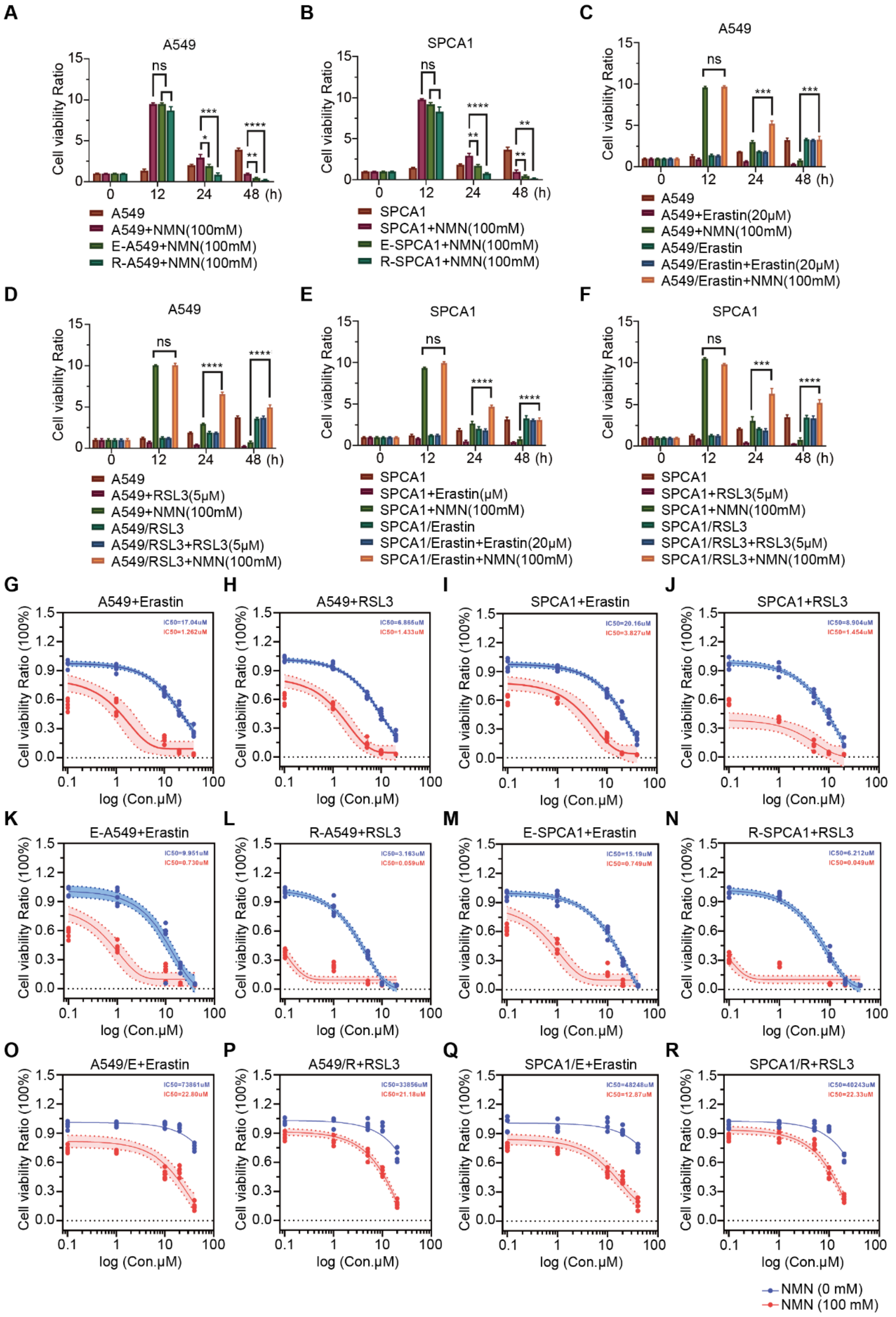

Supplementary Figure S2. NMN accelerated lung adenocarcinoma cells death under ferroptosis inducers treatment

Pretreatment of A549 and SPCA1 cells with ferroptosis inducer Erastin (or RSL3)

increased the sensitivity of cells to Erastin (or RSL3), and the cells were subsequently named E-A549 and E-SPCA1 (or R-A549 and R-SPCA1). Consistently, A549 and SPCA1 cells were induced to be resistant to Erastin (or RSL3), named A549/E and SPCA1/E (or A549/R and R-SPCA1/R). **A-F,** Cell proliferation of A549, E-A549, A549/E, R-A549, and A549/R (SPCA1, E-SPCA1, APCA1/E, R-SPCA1, SPCA1/R) were measured by CCK8 assay in indicated groups (n = 5). **G-R,** Nonlinear regression dose-response IC50 of A549, E-A549, A549/E, R-A549, and A549/R (SPCA1, E-SPCA1, APCA1/E, R-SPCA1, and SPCA1/R) cells were measured by CCK8 assays.
